# Supplementary material for: MiR-23b and miR-133 Cotarget TGFβ2/NOTCH1 in Sheep Dermal Fibroblasts, Affecting Hair Follicle Development
Source: Cells. 2024 Mar 21;13(6):557. doi: 10.3390/cells13060557 (PMC10969380; doi:10.3390/cells13060557)
Supplement: Supplementary file 1 [file cells-13-00557-s001.zip › Supplementary Files/Additional file 5-Figure S5.pdf]

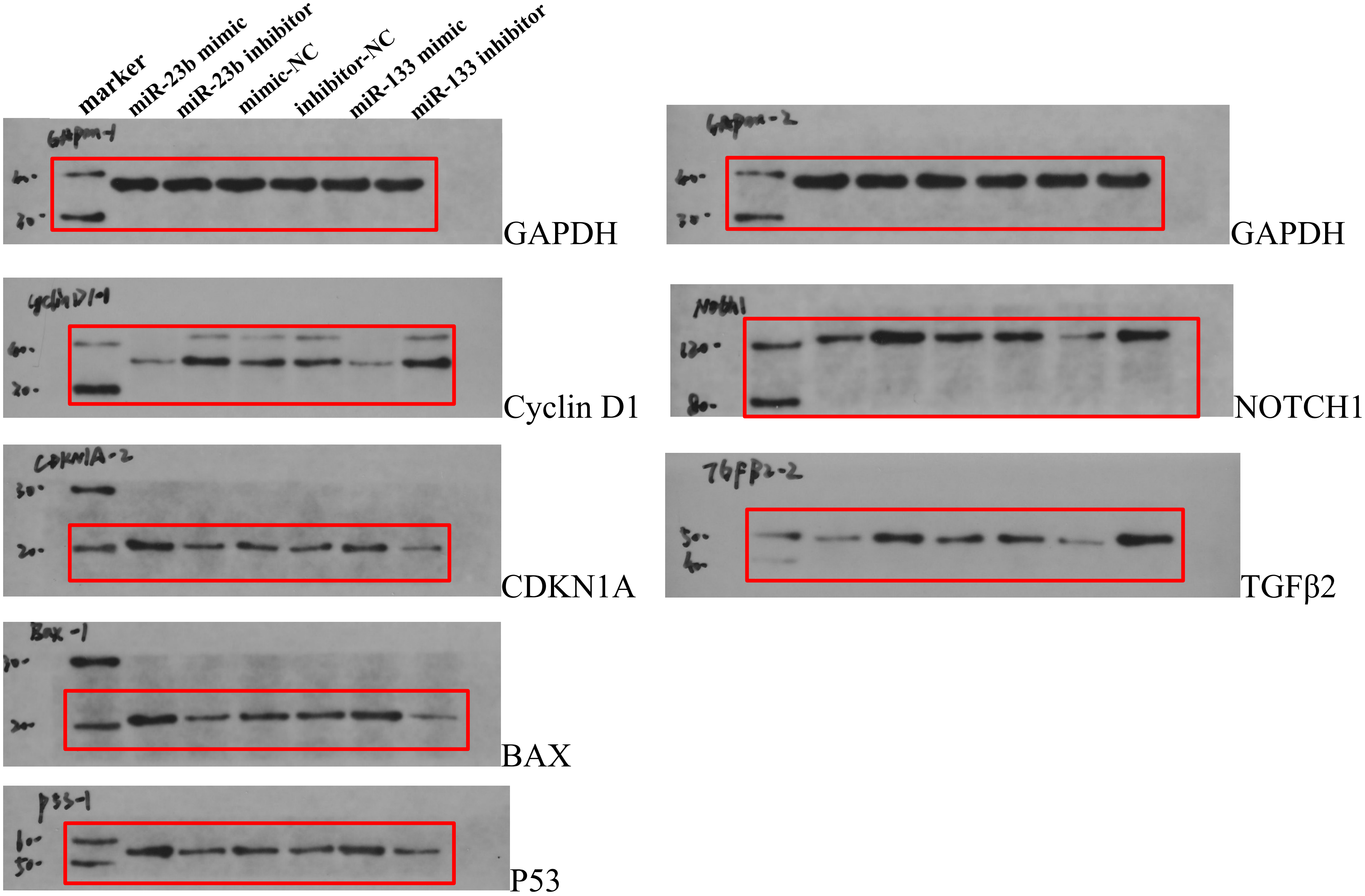

Figure S5. Original image of Western blotting. The order of loading is **marker, miR-23b mimic, miR-23b inhibitor, mimic-NC, inhibitor-NC, miR-133 mimic, miR-133 inhibitor**. The red box represents the part of the manuscript where the images are displayed.
