# Supplementary figures and images for: MiR-23b and miR-133 Cotarget TGFβ2/NOTCH1 in Sheep Dermal Fibroblasts, Affecting Hair Follicle Development
Source: Cells. 2024 Mar 21;13(6):557. doi: 10.3390/cells13060557 (PMC10969380; doi:10.3390/cells13060557)

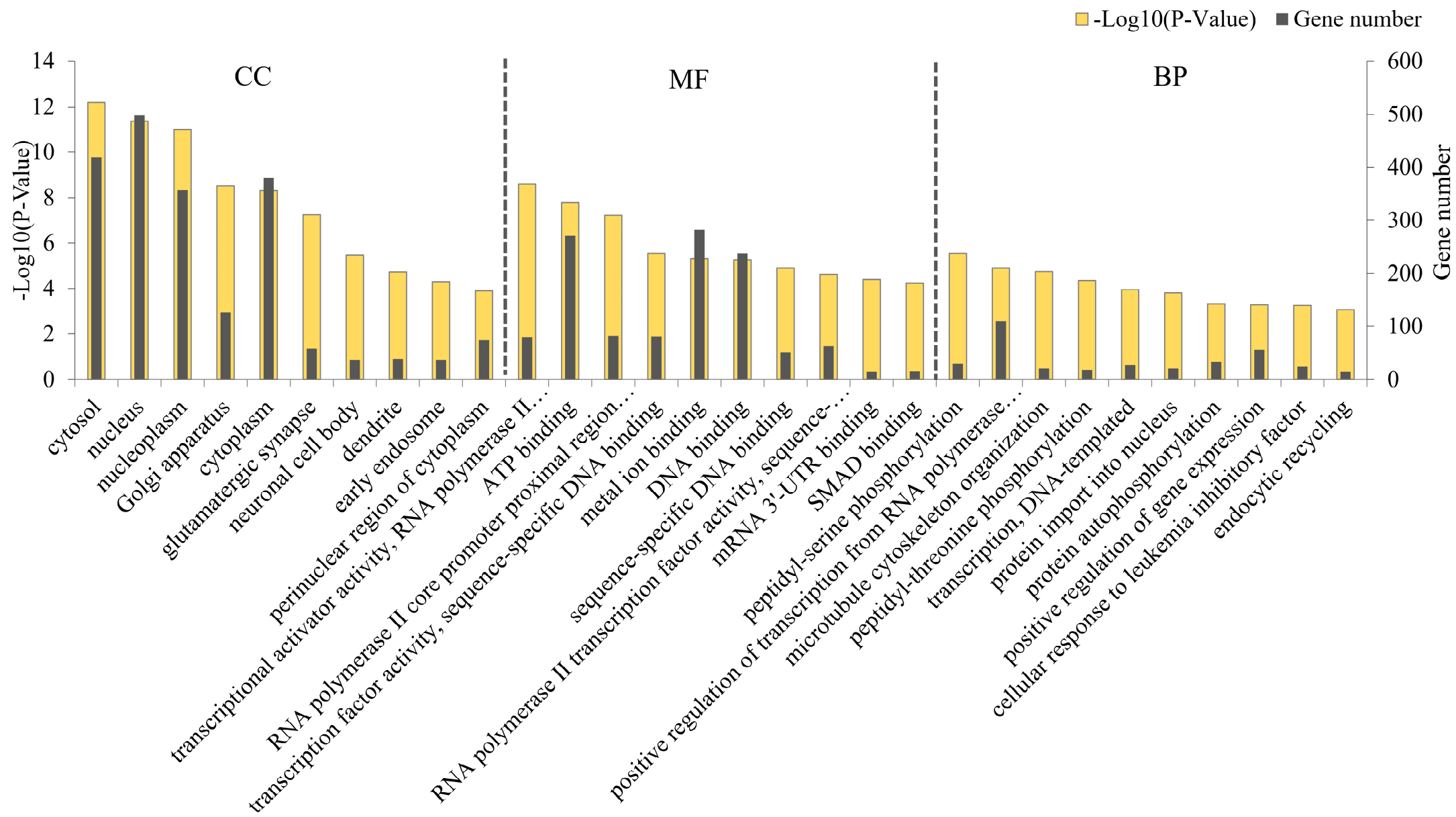

Figure S1. oar-miR-23b target genes top 10 GO functional enrichment histogram.

Supplement: Supplementary file 1 [file cells-13-00557-s001.zip › Supplementary Files/Additional file 1-Figure S1.pdf]

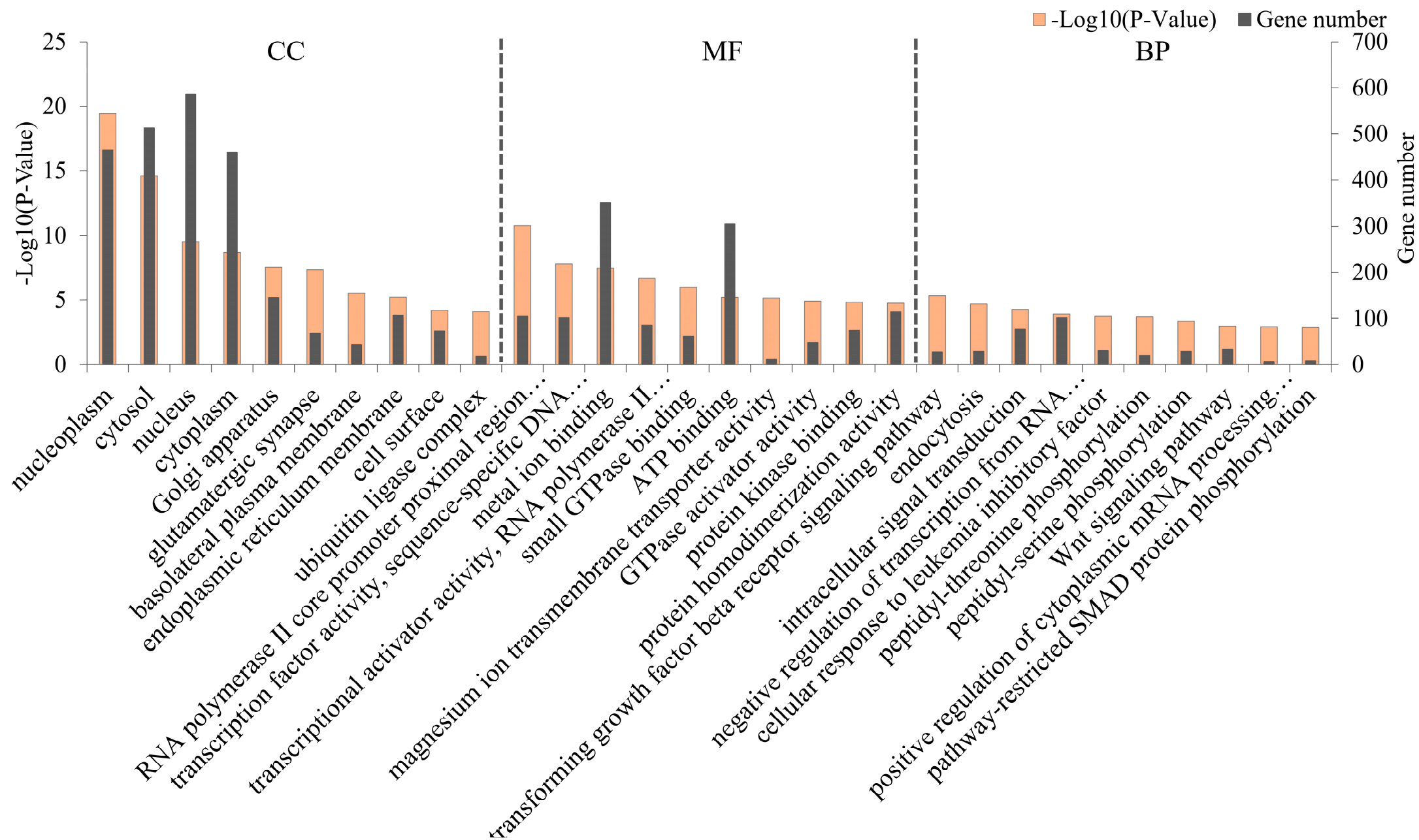

Figure S2. oar-miR-133 target genes top 10 GO functional enrichment histogram.

Supplement: Supplementary file 1 [file cells-13-00557-s001.zip › Supplementary Files/Additional file 2-Figure S2.pdf]

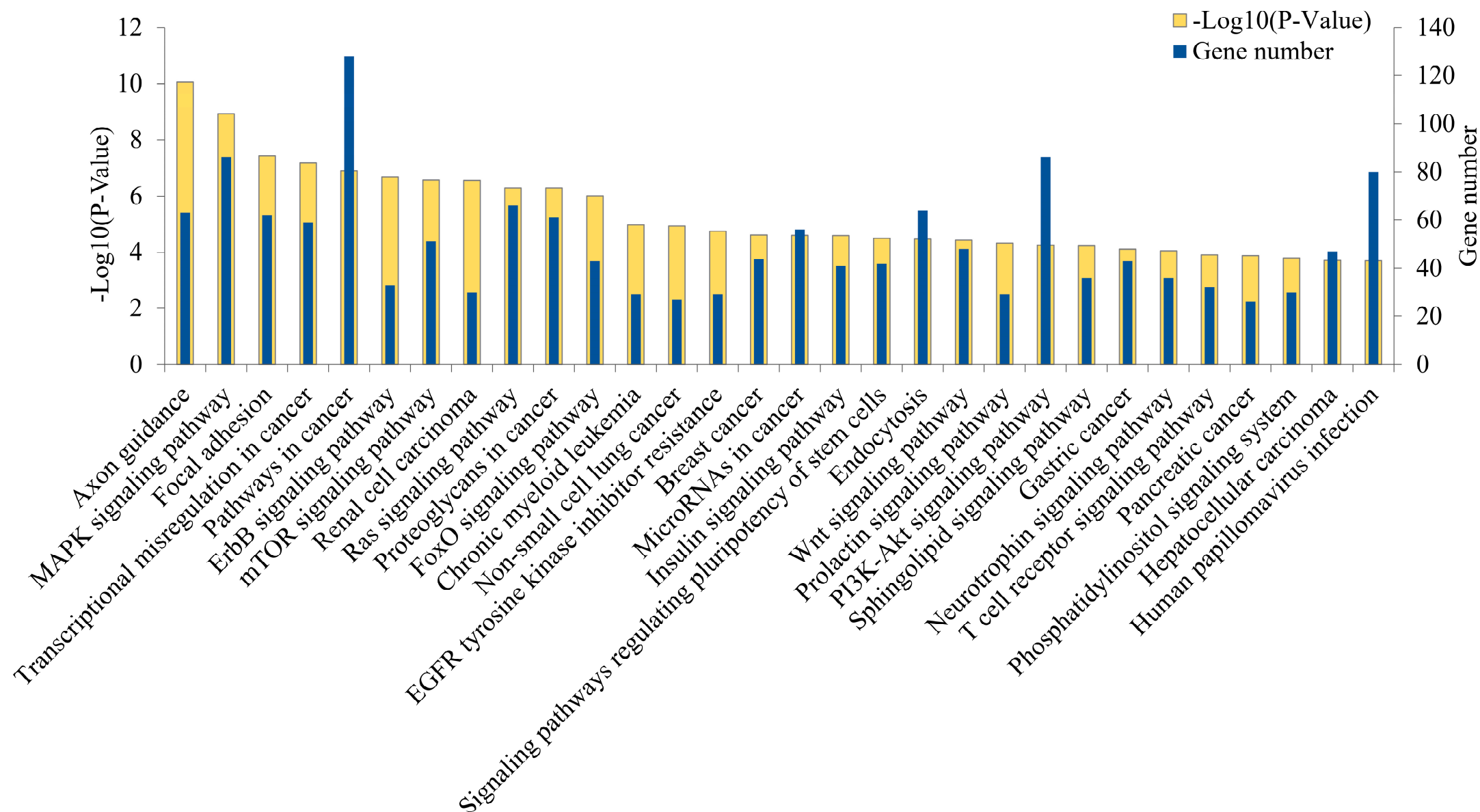

Figure S3. oar-miR-23b target genes top 30 KEGG functional enrichment histogram.

Supplement: Supplementary file 1 [file cells-13-00557-s001.zip › Supplementary Files/Additional file 3-Figure S3 .pdf]

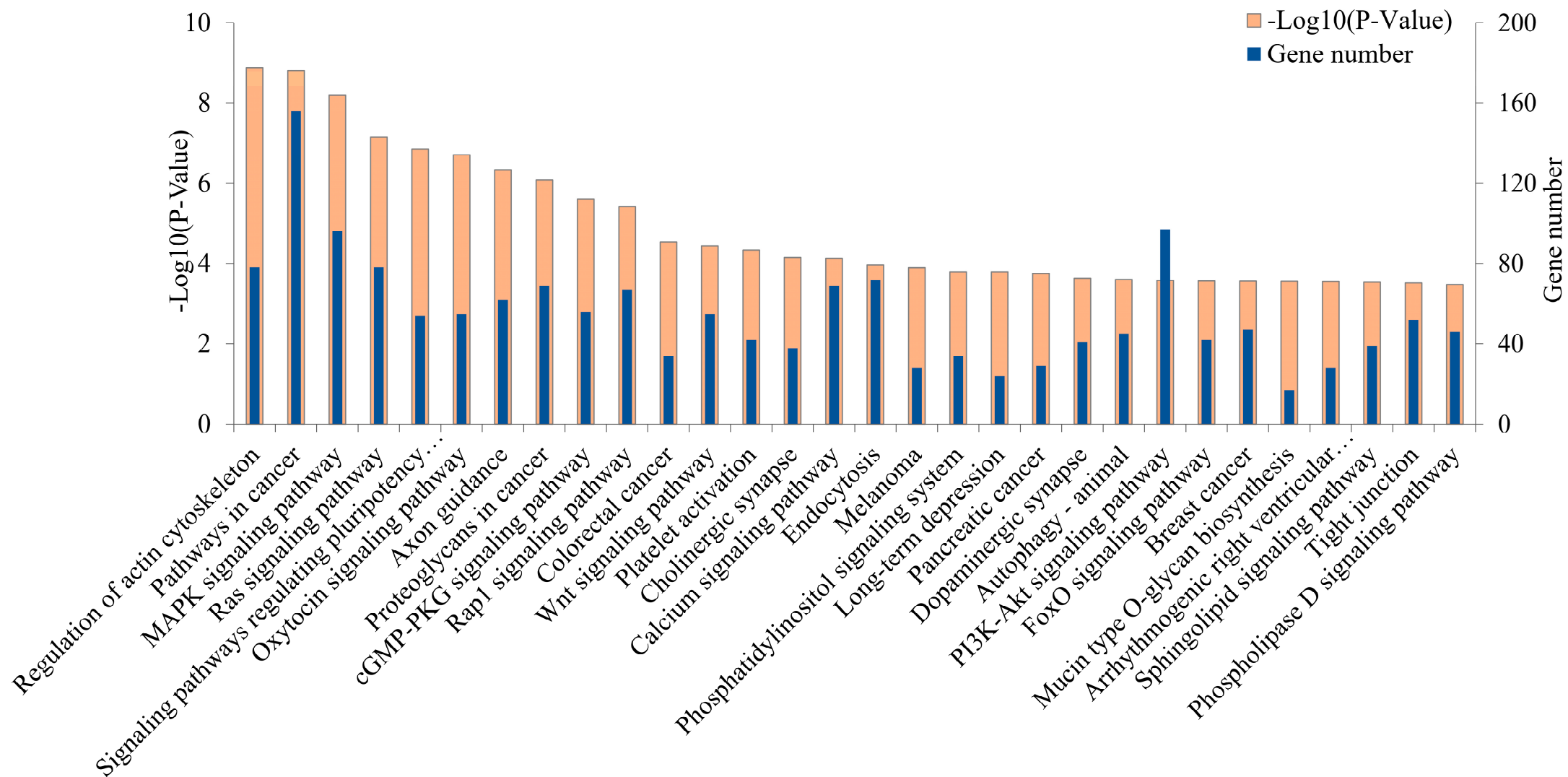

Figure S4. oar-miR-133 target genes top 30 KEGG functional enrichment histogram.

Supplement: Supplementary file 1 [file cells-13-00557-s001.zip › Supplementary Files/Additional file 4-Figure S4.pdf]
